# Warm Pre-Strain: Strengthening the Metastable 304L Austenitic Stainless Steel without Compromising Its Hydrogen Embrittlement Resistance

**DOI:** 10.3390/ma10111331

**Published:** 2017-11-21

**Authors:** Yanfei Wang, Zhiling Zhou, Weijie Wu, Jianming Gong

**Affiliations:** 1 School of Chemical Engineering & Technology, China University of Mining and Technology, Xuzhou 221116, China; 283375421@163.com; 2 School of Mechanical and Power Engineering, Nanjing Tech University, Nanjing 211816, China; stephen_nj@163.com (W.W.); gongjm@njtech.edu.cn (J.G.)

**Keywords:** hydrogen embrittlement, austenitic stainless steels, pre-strain, martensite transformation

## Abstract

Plastic pre-strains were applied to the metastable 304L austenitic stainless steel at both room temperature (20 °C) and higher temperatures (i.e., 50, 80 and 100 °C), and then the hydrogen embrittlement (HE) susceptibility of the steel was evaluated by cathodically hydrogen-charging and tensile testing. The 20 °C pre-strain greatly strengthened the steel, but simultaneously significantly increased the HE susceptibility of the steel, since α′ martensite was induced by the pre-strain, causing the pre-existence of α′ martensite, which provided “highways” for hydrogen to transport deep into the steel during the hydrogen-charging. Although the warm pre-strains did not strengthen the steel as significantly as the 20 °C pre-strain, they retained the HE resistance of the steel. This is because the higher temperatures, particularly 80 and 100 °C, suppressed the α′ martensite transformation during the pre-straining. Pre-strain at a temperature slightly higher than room temperature has a potential to strengthen the metastable 304L austenitic stainless steel without compromising its initial HE resistance.

## 1. Introduction

Due to good hydrogen embrittlement (HE) resistance, austenitic stainless steels (ASSs) have been widely used in the hydrogen-containing environments of chemical and petrochemical industries. Nowadays, they are further considered as one of the candidate materials for pressure vessels or liners storing and transporting gaseous or liquid hydrogen for the upcoming hydrogen energy industry [1,2,3]. However, the ASSs can be embrittled by hydrogen with varying degree seemingly depending on their stability against strain-induced α′ martensite transformation. That is, the stable ASSs, e.g., 310S, in which no α′ martensite forms during strain, are found less sensitive to hydrogen [3,4,5], while the metastable ones, e.g., 304L, which undergo the transformation from γ austenite to α′ martensite during strain, can be severely embrittled by hydrogen [3,4,6,7,8,9,10]. Although the exact role of strain-induced α′ martensite in the HE mechanism of ASSs is still unclear (e.g., is the austenite itself embrittled or is the strain-induced α′ martensite embrittled), many researchers have accepted that the induced α′ martensite can provide “highways” for hydrogen transport in the ASSs [7,8,9], consequently increase their HE susceptibility.

ASS components, e.g., vessels and liners, often inevitably suffer from some degree of plastic deformation during fabrication, such as tension, bending, stretching and drawing. Most of these deformations are carried out at room temperature. Moreover, since the ASSs at the solution-annealed state possess excellent plasticity but lower yield strength, cold-stretching technique [11,12,13] has been applied to them purposely to elevate their strength based on the concept of strain-strengthening (work-hardening). For example, for a pressure vessel, the cold-stretching technique is generally performed at room temperature by pressurizing the vessel to a pressure known to produce required amount of plastic deformation [12]. The vessel then becomes stronger due to strain-strengthening to withstand a service pressure with reduced wall thickness, hence saving the materials and reducing the weight. The cold-stretching technique has been involved in several standards, e.g., EN 13458-2 Appendix C [14], AS 1210 Supplement 2 [15], and ASME Code Case 2596 [16]. However, the prior plastic deformations can induce α′ martensite transformation in the metastable ASSs, causing the pre-existence of α′ martensite before hydrogen exposure. Many studies have shown that the pre-existing α′ martensite can enhance the hydrogen transport and thus decrease the HE resistance of ASSs. For instance, Perng and Altstetter [17] showed that, in the metastable 301 and 304 steels, the diffusivity and permeability of hydrogen were greatly enhanced by increasing pre-strain-induced α′ martensite. Mine et al. [18] found that the fatigue crack growth rate was more sensitive to hydrogen in the pre-strained 304 steels since α′ martensite had been created by the pre-strain. On the contrary, the more stable ASSs were found to be less affected by the pre-strain, since little or no α′ martensite was induced by the pre-strain [17,18]. Martin et al. [19] revealed a detrimental effect of machining-induced α′ martensite on the performance of 304 steel in hydrogen. Wang et al. [20] found that, since the volume fraction of induced α′ martensite increased with increasing pre-strain, a higher level of pre-strain resulted in higher hydrogen diffusivity and more severe HE in the 304L steel. Thus, it is indicated that, when applying the cold pre-strain to the metastable ASSs, more attention should be paid to its negative effect on HE resistance due to strain-induced α′ martensite transformation.

It is known that the transformation from γ austenite to α′ martensite in the metastable ASSs depends on the temperature at which the strain is applied. For the most widely-used 304 and 304L ASSs, it is found that, pre-straining at the temperatures close to room temperature can induce α′ martensite transformation in the steels, but elevating the pre-strain temperature can make the transformation less severe [21,22,23]. Considering that, a higher temperature can suppress the α′ martensite transformation in the 304L ASS, we are motivated to examine whether we can apply the prior plastic deformation, or the cold-stretching technique, to the metastable 304L ASS at a temperature slightly higher than room temperature, without inducing severe α′ martensite transformation, thus preserving its original good HE resistance. In fact, a pre-strain at a slightly elevated temperature can still be able to strengthen the metastable ASSs. Therefore, in this paper, we pre-strained the metastable ASS 304L by tension at various temperatures, i.e., 20 (room temperature), 50, 80 and 100 °C, and then evaluated its HE susceptibility by cathodically hydrogen pre-charging and tensile testing.

## 2. Materials and Experimental

The 304L steel used has a chemical composition of Fe-0.02C-0.37Si-1.15Mn-0.004S-0.031P-18.3Cr-8.1Ni-0.044N-0.014Mo-0.064Cu, wt %. It was received in the form of a 10 mm thick plate and was solution-annealed at 1080 °C for 1 h and water quenched. The Md_30_ temperature, i.e., the temperature at which tensile straining to 30% induces 50% volume fraction of α′ martensite, is calculated to be 21 °C according to the formula proposed by Nohara et al. [24]: Md_30_ (°C) = 551 − 462(C + N) − 9.2Si − 8.1Mn − 29(Ni + Cu) − 13.7Cr − 18.5Mo (wt %), confirming that pre-straining at room temperature may induce α′ martensite transformation in the steel. Tensile specimens with a gauge size of 25(length) × 10(width) × 2(thickness) mm^3^ were cut from the plate with their length direction parallel to the rolling direction. The specimen surfaces were ground with successive grades of emery paper up to 2000 grit, polished with paste, washed with deionized water and dried. Tensile plastic pre-strains were applied to the specimens along the length direction by tension at 20, 50, 80 and 100 °C, respectively, at a strain rate of $2.5\times 10^{-4}{\;s}^{-1}$ using a tensile testing machine with a heating furnace. The specimens are firstly heated to the required temperature slowly (~5 °C/min) and then they were clamped and pre-strained. The plastic pre-strain level was set as 30 ± 2% (nominal strain), as this strain level is high enough to cover the strain range usually used in the cold-stretching technique.

To identify the α′ martensite transformation, the microstructures of both un-pre-strained and pre-strained specimens were examined by an optical microscope (OM) after etching by the Beraha’s reagent (0.5 g potassium metabisulfite, 20 mL HCl and 100 mL distilled water) according to [25]. The phase identification and quantitative estimate of phase volume fractions were further determined by the X-ray diffraction (XRD) technique. The XRD measurements were conducted with a 2θ range from 40° to 100° at room temperature using a diffractometer (Rigaku SmartLab, Tokyo, Japan) with a Cu-Kα (λ = 0.154056 nm) radiation operating at 40 kV and 30 mA at a step size of 0.02°. The quantitative estimation of phases by XRD is based on the principle that the total integrated intensity of all diffraction peaks for each phase in a mixture is proportional to the volume fraction of that phase [22,26,27]. For an ASS containing γ austenite (fcc), α′ martensite (bcc) and ε martensite (hcp), by considering the (220)_γ_, (311)_γ_, (111)_γ_, and (200)_γ_ reflections for γ austenite, (200)_α′_, (211)_α′_ and (110)_α′_ reflections for α′ martensite, and (101)_ε_ and (102)_ε_ for ε martensite, the volume fraction of austenite and martensite can be derived from the numerous peaks by the following formula:

(1) $$V_{i}=\frac{\frac{1}{n}\sum_{j=1}^{n}\frac{I_{i}^{j}}{R_{i}^{j}}}{\frac{1}{n}\sum_{j=1}^{n}\frac{I_{\gamma }^{j}}{R_{\gamma }^{j}}+\frac{1}{n}\sum_{j=1}^{n}\frac{I_{\alpha^{\prime }}^{j}}{R_{\alpha^{\prime }}^{j}}+\frac{1}{n}\sum_{j=1}^{n}\frac{I_{\varepsilon }^{j}}{R_{\varepsilon }^{j}}}$$

where *i* = γ, α′ or ε, *n* is the number of peaks of the phase examined, *I_i_* and *R_i_* are the integrated intensity of reflecting plane and material scattering factor, respectively. Each *R_i_* value used was obtained from Ref. [26]. In our previous study [28], we have used the magnetic induction method (the α′ martensite is ferromagnetic) to verify the XRD method used, and found that the two methods are comparable in detecting the presence of α′ martensite.

The specimens were then cathodically charged with hydrogen in 0.1 mol/L NaOH solution at a current density of 1.1 $\mathrm{mA}/{\mathrm{cm}}^{2}$ for 48 h at 50 °C. When the hydrogen-charging was completed, the specimens were taken to tensile testing in air within 5 min also at the strain rate of $2.5\times 10^{-4}{\;s}^{-1}$ by the tensile testing machine. Two replicated specimens were used for each testing condition. The specimens un-charged were also tested as references. Tensile curves and properties were obtained. The HE index, namely, the plasticity loss

(2) $$\delta_{L}=\left(\delta_{0}-\delta_{H}\right)/\delta_{0}$$

where $\delta_{0}$ and $\delta_{H}$ are the elongation at fracture of un-charged and charged specimens, respectively, was used to quantitatively evaluate the HE susceptibility. The HE susceptibility increases with increasing $\delta_{L}$. The fracture surfaces of broken specimens were examined by a scanning electron microscope (SEM, JEOL JSM-6510, JEOL Ltd., Tokyo, Japan). In addition, an extra group of specimens was pre-strained and hydrogen-charged, and then the total hydrogen amounts charged into the specimens by the hydrogen-charging were determined by the inert gas impulse fusion heat conductivity method using a hydrogen-oxygen-nitrogen analyzer (LECO ONH836, LECO Corporation, St. Joseph, MI, USA).

## 3. Results and Discussion

**Figure 1:**
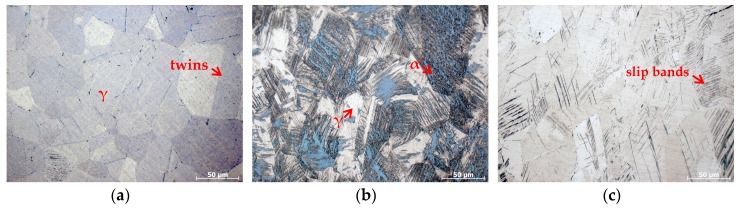
Microstructures of specimens: (**a**) Un-pre-strained; (**b**) 30% pre-strained at 20 °C; (**c**) 30% pre-strained at 80 °C.

**Figure 2:**
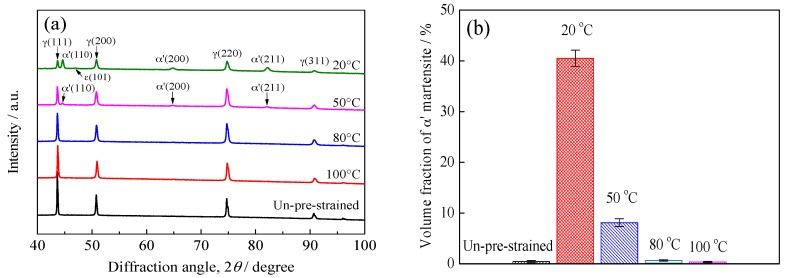
X-ray diffraction (XRD) results of specimens: (**a**) The XRD patterns; (**b**) The volume fraction of α′ martensite.

The OM microstructures and XRD results are shown in Figure 1 and Figure 2, respectively. The un-pre-strained specimens have a typical microstructure of solution-annealed ASSs, i.e., composed of nearly-equiaxed grains of austenite and annealing twins, see Figure 1a, and no α′ martensite diffraction peak was detected (a ε martensite peak with a lower intensity was detected), see Figure 2a, indicating an almost full single-phase austenite microstructure. In contrast, the specimens pre-strained at 20 °C exhibit severe strain-induced α′ martensite transformation, as indicated in Figure 1b by the etched dark regions [22,25,27] and Figure 2a by the presence of great α′ martensite diffraction peaks. Figure 2b shows an average volume fraction of α′ martensite of 40.5% in the 20 °C pre-strained specimens, indicating that nearly half of the specimens were transformed into α′ martensite. However, the specimens pre-strained at 50 °C suffered from less severe α′ martensite transformation, as indicated in Figure 2a by the lower intensity of α′ martensite diffraction peaks and Figure 2b by the volume fraction of α′ martensite of 8.1%. Furthermore, it is found that the specimens pre-strained at 80 and 100 °C demonstrated almost no strain-induced α′ martensite transformation, see Figure 1c and Figure 2. Thus, the results clearly indicate that the higher temperatures can effectively suppress the α′ martensite transformation during the pre-strain, in agreement with [21,22,23], and the higher the temperature at which the pre-strain is applied the less severe the α′ martensite transformation is induced. In addition, in these pre-strained specimens, parallel or intersecting slip bands were also shown, evdicing the occurance of palstic deformation, see Figure 1.

**Figure 3:**
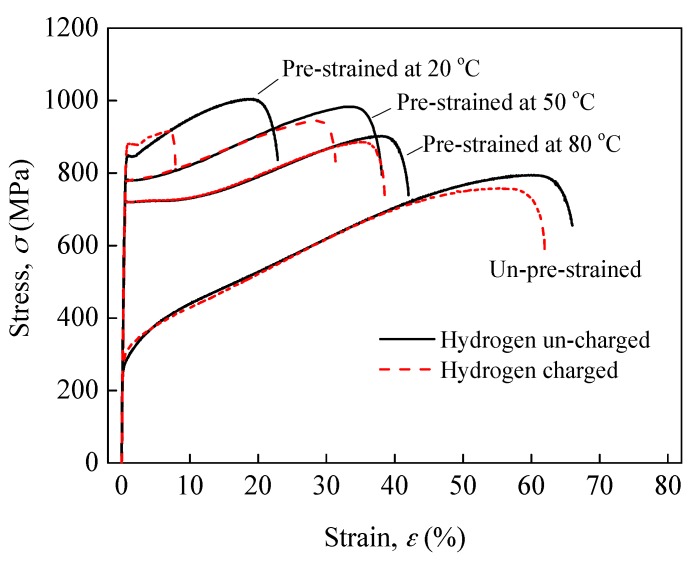
Nominal stress-strain curves of specimens.

Figure 3 shows the representative stress-strain curves. The 30% pre-strain significantly elevated the yield strength and tensile strength but reduced the elongation at fracture of the un-charged specimens, particularly at 20 °C, indicating that the strain-strengthening of 304L steel depends on the strain temperature. For example, after 30% pre-strain at 20 °C, the yield strength increased from 265 to 850 MPa by 3.2 times. At 50 °C, it increased to 785 MPa by 3 times and at 80 °C to 720 MPa by 2.7 times. The mechanism of strain-strengthening of ASSs has been well-established in literatures, e.g., [22,29,30]. It should be noted that, for the metastable ASSs, the strain-induced α′ martensite transformation also plays a role, since not only it is inherently harder but also it can induce an excess increase in dislocation density to accommodate the volume expansion and can act as obstacles for slip. Thus, the strain-strengthening at room temperature was more significant due to the α′ martensite transformation. However, it can be found that the gaps between these strength values for the three temperatures are marginal, indicating that pre-straining the steel warmly, i.e., at a temperature slightly higher than room temperature, can still strengthen the 304L steel by a very large degree, although the α′ martensite transformation is suppressed.

**Figure 4:**
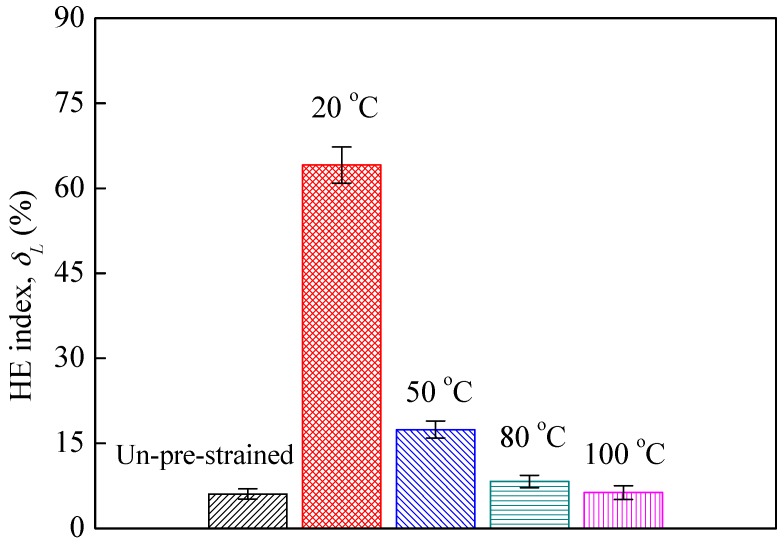
Hydrogen embrittlement (HE) index of hydrogen-charged specimens.

**Figure 5:**
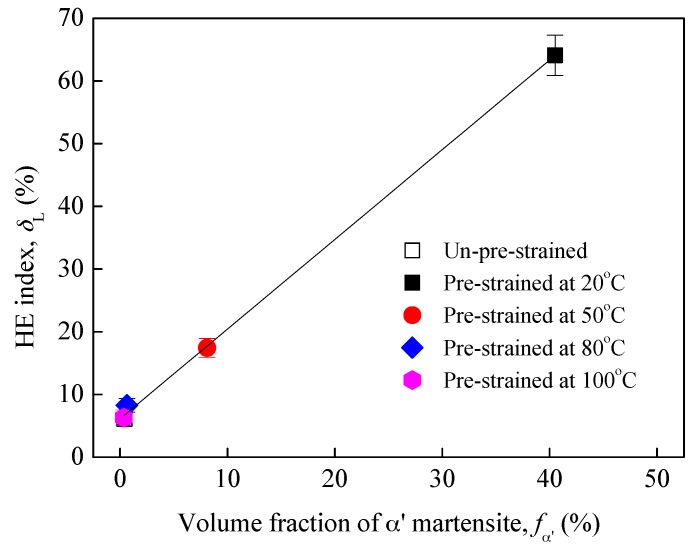
HE index as a function of pre-existing α′ martensite.

Figure 3 shows that the hydrogen-charging had limited influence on the yield strength of all specimens, but decreased the tensile strength and elongation at fracture, indicating a loss in plasticity due to hydrogen-charging, i.e., HE. Figure 4 plots the average HE index $\delta_{L}$ calculated for the specimens. The $\delta_{L}$ of un-pre-strained specimens is 6.1%, which is very low indicating the good HE resistance of 304L steel without pre-strain, i.e., at the solution-annealed state, whereas after pre-strain at 20 °C, the $\delta_{L}$ was increased by more than 10 times, i.e., was increased to 64.1%, meaning that the 20 °C pre-strain significantly increased the HE susceptibility of the 304L steel. Clearly, this significant increase in HE susceptibility can be preferentially related to the pre-existence of a large volume fraction of α′ martensite induced by the 20 °C pre-strain, as noted by the authors [16,17,18,19]. However, for the specimens pre-strained at 50 °C, the $\delta_{L}$ is 17.4%, and for the specimens pre-strained at 80 and 100 °C, the $\delta_{L}$ are 8.2% and 6.3%, respectively, indicating that the warm pre-strains did not enhance the HE susceptibility of the 304L steel, particularly the 80 and 100 °C pre-strains. This result can be attributed to that the higher temperatures had suppressed the α′ martensite transformation during the pre-strain. Figure 5 depicts the HE index $\delta_{L}$ as a function of pre-strain-induced α′ martensite volume fraction. Evidently, regardless of the pre-strain temperature, the HE susceptibility of the steel almost linearly increases with increasing pre-existing α′ martensite amount.

**Figure 6:**
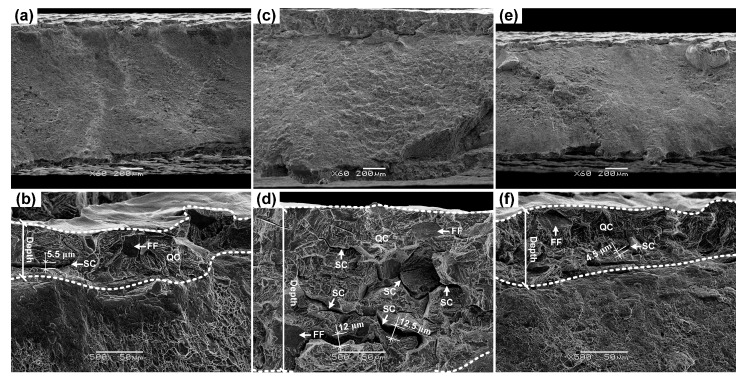
SEM fracture surfaces of broken specimens. (**a**,**b**) Un-pre-strained; (**c**,**d**) Pre-strained at 20 °C; (**e**,**f**) Pre-strained at 80 °C.

Turning to the SEM morphologies of the fracture surfaces, all specimens without hydrogen-charging exhibit dimples on their whole fracture surfaces, revealing a ductile fracture. However, all hydrogen-charged specimens show two zones with different fracture features, i.e., the central regions show dimples, but the edge regions, where the materials were exposed to hydrogen during hydrogen-charging, show transgranular quasi-cleavage (QC) fracture with some flat facets (FFs) and secondary cracks (SCs). As examples, Figure 6 shows the results of specimens un-pre-strained and pre-strained at 20 and 80 °C, respectively. In fact, this fracture feature of edge regions has been well-established as the typical mode of hydrogen-induced brittle fracture in hydrogenated metastable ASSs [6,7,8,9]. The formation of flat facets can be related to the twin boundary separations [6,7,8,9]. However, the SCs in the 20 °C pre-strained specimens are more and bigger (wider and longer), indicating a more severe HE. For example, the widths of some SCs presenting in the 20 °C pre-strained specimens, see Figure 6d, exceed 10 μm, but the widths of the SCs in the un-pre-strained and 80 °C pre-strained specimens, see Figure 6b,f, do not exceed 6 μm.

It is found that the depth of brittle fracture regions varies with the pre-strain temperature. The 20 °C pre-strained specimens have a largest brittle region depth. The depth of brittle fracture region can roughly represent the maximum distance of hydrogen transporting into the specimen during the hydrogen exposure, thus it can be used to approximately evaluate the apparent hydrogen diffusivity. For the plate-type specimen, according to Fick’s law, the distribution of hydrogen along the depth direction during charging can be formalized by [18,31,32,33]

(3) $$c\left(x,t\right)=c_{i}+\left(c_{s}-c_{i}\right)\left[1-\mathrm{erf}\left(\frac{x}{2\sqrt{Dt}}\right)\right]$$

where x is the depth from the specimen surface; $c_{i}$ is the initial hydrogen concentration in the specimen, which is taken as zero here since the hydrogen amount measurement results show a negligible hydrogen amount in the un-charged specimens; $c_{s}$ is the hydrogen concentration dissolved at the specimen surface during the charging; *D* is the apparent diffusivity of hydrogen; *t* is the charging time. Since when $x/2\sqrt{Dt}\geq 2$, c approaches to 0, the maximum hydrogen transport distance $x_{\max}$ can be determined approximately by taking $x_{\max}=4\sqrt{Dt}$ [20,31,32]. Based on this equation, the *D* is calculated as $7.6\times 10^{-16}{\;m}^{2}/s$ for the un-pre-strained specimens (this value is in good agreement with those reported previously in [2,17,33] for solution-annealed ASSs, i.e., $1.8-8.0\times 10^{-16}$ $m^{2}/s$), and $1.7\times 10^{-14}$, $2.8\times 10^{-15}$, $10.1\times 10^{-16}$, $9.4\times 10^{-16}{\;m}^{2}/s$ for the 30% pre-strained specimens at 20, 50, 80 and 100 °C, respectively. It is indicated that, compared with the un-pre-strained specimens, the 30% pre-strain at 20 °C increased the diffusivity by about 22 times, and the 30% pre-strain at 50 °C increased the diffusivity by 4 times, while the 30% pre-strain at 80 and 100 °C had almost no influence on the diffusivity. This result corresponds to that a large amount of α′ martensite had formed in the 20 °C pre-strained specimens, while only a little α′ martensite had formed in the 50 °C specimens and almost no α′ martensite had formed in the 80 and 100 °C specimens. It has been established that the pre-existing α′ martensite induced by pre-strain in ASSs can provide “highways” for hydrogen diffusion and transport [7,8,9]. Since there was a large volume fraction of α′ martensite pre-existing in the 20 °C pre-strained specimens, the apparent diffusivity of hydrogen was markedly increased. Consequently, the hydrogen transported into the specimens by a very deep depth during hydrogen-charging, resulting in severe HE for the 20 °C pre-strained specimens. There was only 8.1% α′ martensite pre-existing in the 50 °C pre-strained specimens, thus the specimens have a HE susceptibility just slightly higher than the un-pre-strained specimens. For the specimens pre-strained at 80 and 100 °C, since the α′ martensite was almost completely suppressed, the specimens still mainly composed of single-phase austenite after pre-straining, consequently the diffusivity of hydrogen was nearly unchanged, and the specimens retained its good resistance to the HE.

**Figure 7:**
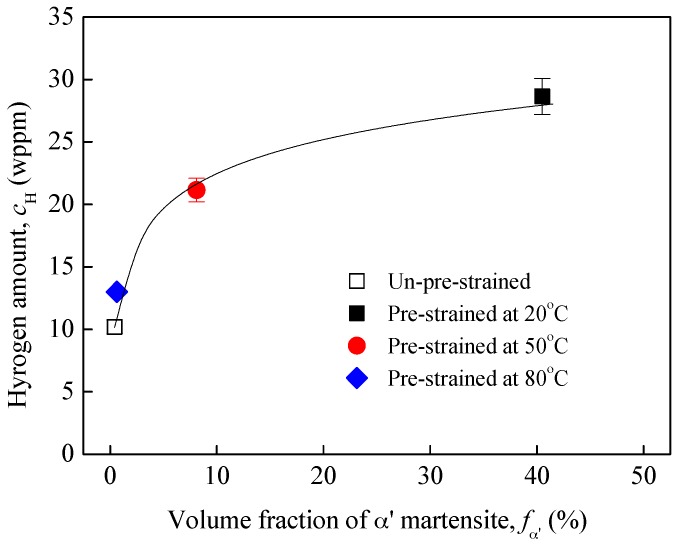
Total hydrogen amounts charged into the steel as a function of pre-existing α′ martensite.

The total hydrogen amounts charged into the specimens are shown in Figure 7 as a function of pre-strain-induced α′ martensite. The results confirm that the α′ martensite can provide “highways” for the hydrogen to transport into the specimens. There was a large volume fraction of α′ martensite in the 20% pre-strained specimens, and the diffusivity of hydrogen in the specimens was increased, thus the total hydrogen amount transporting into the specimens was very high. Moreover, regardless of pre-strain temperature, with increasing volume fraction of pre-strain-induced α′ martensite, the total hydrogen amount charged into the specimens increased. However, it should be noted that this increase became less significant with increasing α′ martensite, this is because the α′ martensite possesses lower hydrogen solubility. The increase in α′ martensite resulted in a decrease in apparent hydrogen solubility, thus the total hydrogen amounts charged into the specimens decreased.

The results show that, the 50, 80 and 100 °C pre-strains can still strengthen the 304L steel by a very large degree, thus it can be concluded that the warm pre-strain has a potential to strengthen the metastable ASSs without compromising their excellent HE resistance before pre-strain, i.e., at the solution-annealed state. For example, for an ASS pressure vessel to be exposed to a hydrogen-containing environment during service, e.g., high pressure gaseous hydrogen, if we pressurize it warmly, i.e., at a temperature slightly higher than room temperature, e.g., 80 °C for the 304L steel, during the application of “cold”-stretching technique, on one hand, we can still strengthen the vessel (although the strengthening effect is not as significant as the stretching at room temperature or lower temperature), consequently using reduced wall thickness and weight to withstand the pressure, on the other hand, we can still expect a higher HE resistance for the vessel, as the α′ martensite transformation is suppressed by the warm-stretching.

## 4. Conclusions

This paper investigated the effect of 30% tensile pre-strain applied respectively at 20 (room temperature), 50, 80 and 100 °C on the HE resistance of metastable 304L austenitic stainless steel. The main conclusions are as follows:

(1) The 30% pre-strain at 20 °C significantly elevates the strength of 304L steel, whereas it also induces a large volume fraction of α′ martensite in the steel. Since the pre-existing α′ martensite can provide “highways” for the hydrogen to transport into the steel during subsequent hydrogen exposure, the pre-strained steel exhibits severe HE. The HE resistance of 304L steel can be markedly impaired by the room temperature pre-strain.
(2) In the 304L steel, the 30% pre-strain at 50 °C induces a little amount of α′ martensite, and those at 80 and 100 °C induce almost no α′ martensite. Since the α′ martensite transformation is suppressed by the higher temperatures, particularly the 80 and 100 °C, the subsequent HE resistance of 304L steel is not impaired. When pre-straining a metastable ASS, elevating slightly the temperature helps preserve the HE resistance of the steel.
(3) Although the strengthening effect of the pre-strains at 50, 80 and 100 °C is not as significant as the pre-strain at room temperature, i.e., 20 °C, they can still strengthen the 304L steel by a very large degree, thus, we can apply the warm pre-strain, e.g., 80 °C for the 304L steel, to strain-strengthen the metastable ASSs, reducing the weight and cost of components but without compromising their original good HE resistance.
